# Supplementary material for: Factors Contributing to Delayed Return to Work among French Healthcare Professionals Afflicted by COVID-19 at a Hospital in the Rhône-Alpes Region, 2021
Source: Int J Environ Res Public Health. 2023 Oct 26;20(21):6979. doi: 10.3390/ijerph20216979 (PMC10650843; doi:10.3390/ijerph20216979)
Supplement: Supplementary file 1 [file ijerph-20-06979-s001.zip › ijerph-2569913-supplementary.pdf]

Table S1 : Joinpoint regression analysis results

Symptom	Joinpoint day	Slope before joinpoint	Slope after joinpoint
Asthenia	16	-0,042	-0,007
Fever	6	-0,091	-0,002
Headache	11	-0,061	-0,004
Myarthralgia	10	-0,059	-0,005
Respiratory disorders	22	-0,018	0
Digestive disorders	10	-0,022	0
Anosmia	22	-0,020	<-0,001
Anxiety	17	-0,021	0

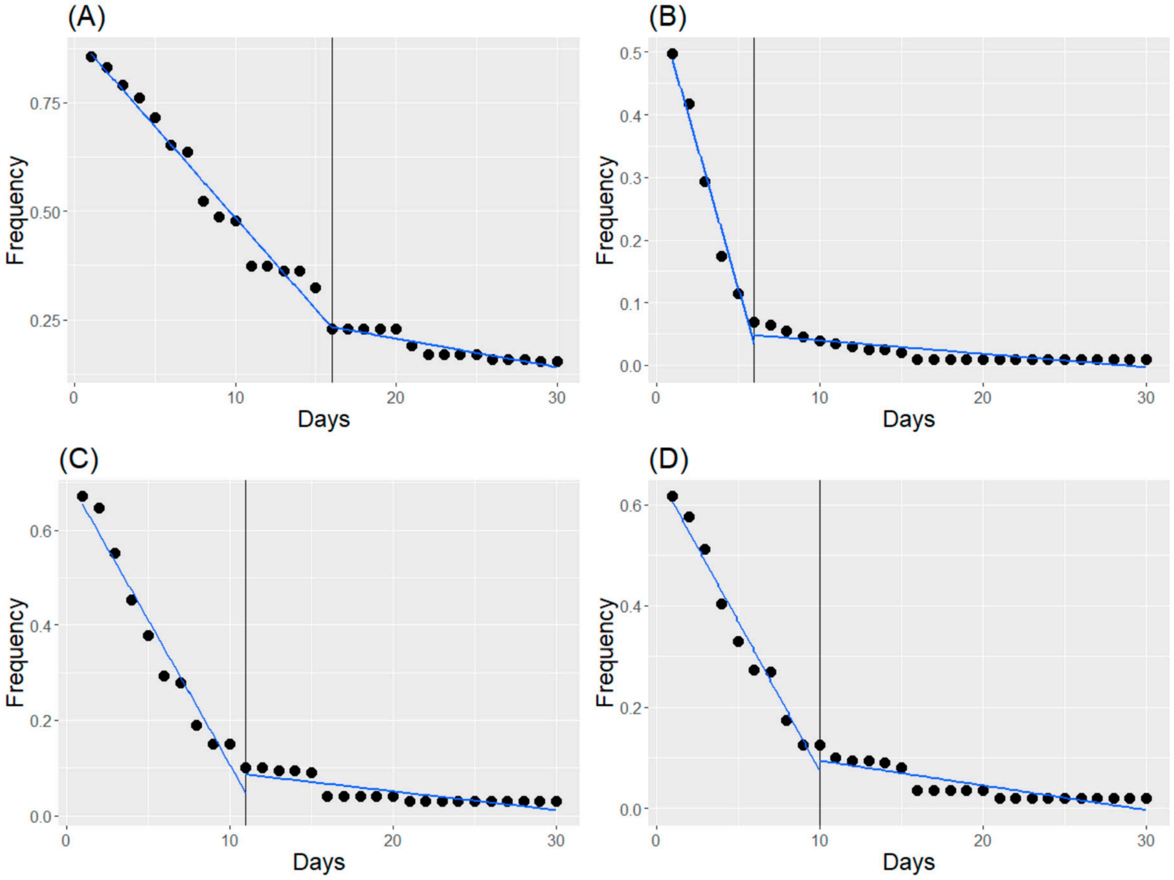

Figure S1 : Symptoms evolution over time
(A) Asthenia frequency over time
(B) Fever frequency over time
(C) Headache frequency over time
(D) Myarthralgia frequency over time

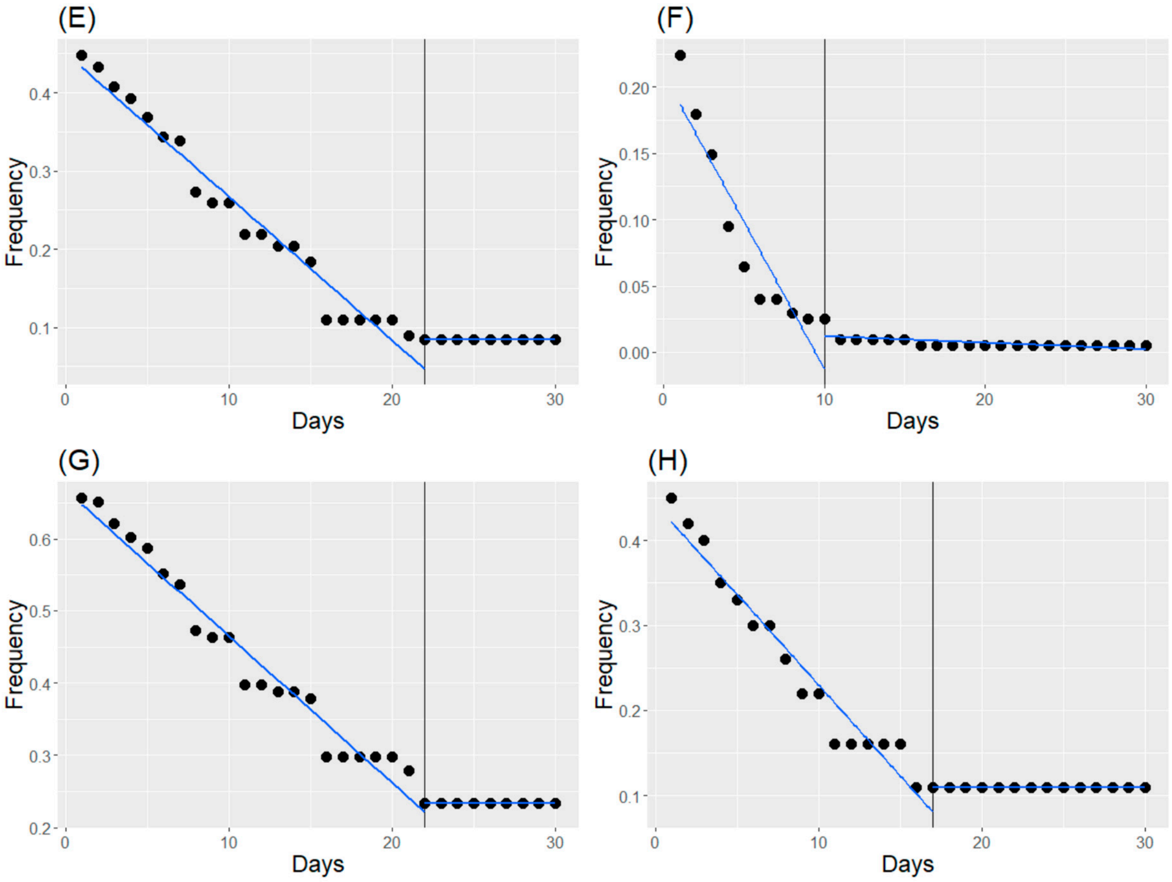

Figure S1bis : Symptoms evolution over time
(E) Respiratory disorders frequency over time
(F) Digestive disorders frequency over time
(G) Anosmia frequency over time
(H) Anxiety frequency over time
